# How much do medical students know about cancer risk factors?

**DOI:** 10.1186/s12909-025-07487-y

**Published:** 2025-07-01

**Authors:** Hamit Sirri Keten, Guler Gizem Dogan, Hatice Tuba Akbayram, Oguz Isik

**Affiliations:** 1 Department of Family Medicine, Medical Faculty, Gaziantep University, Gaziantep, TR-27310 Turkey; 2 Department of Family Medicine, Kilis State Hospital, Kilis, Turkey; 3 Department of Family Medicine, Dr. Aysegül Karakeci Family Health Center, Adana, Turkey

**Keywords:** Cancer, Risk factor, Medical student, Smoking, Knowledge

## Abstract

**Background:**

In this study, we aimed to determine the knowledge level of medical students regarding cancer risk factors (CRF).

**Methods:**

This study was carried out in Gaziantep University (Gaziantep, Turkey) between February 10 and March 04 of 2022. A total of 532 students at Gaziantep University Faculty of Medicine took part in the study. The sociodemographic data of the students and their level of knowledge about CRF were questioned in the survey. Risk status of modifiable CRF (smoking, alcohol, obesity, human papillomavirus, high-fat diet) for cancer types was asked with 36 questions. The maximum possible score was 36 points.

**Results:**

Of the students, 270 (50.8%) were female, 262 (49.2%) were male, and the mean age was 22.63 ± 2.66 years. The mean CRF knowledge score of the participants was 23.17 ± 7.20 out of a maximum of 36. Knowledge scores of women and men were similar (23.06 ± 7.35 vs. 23.32 ± 7.08, respectively *p* = 0.674). The level of knowledge about CRF was significantly lower in preclinical students than in clinical students (*p* < 0.001). The knowledge score of those who received training on CRF was significantly higher than those who did not (*p* < 0.001). The most commonly known CRF was smoking and alcohol. Multivariate linear regression analysis showed that being in clinical years (*p* < 0.001) and having received CRF training (*p* < 0.001) were significant independent predictors of higher knowledge scores. Conclusion: This study indicates that knowledge about CRF among medical students remains suboptimal, with education and clinical exposure playing critical roles in improving scores. It was determined that medical students had serious lack of knowledge about CRF. The knowledge levels of men and women about CRF were similar. Education positively affected knowledge score on CRF, and thus we believe that medical education curricula need to be developed in this area. Educating students about modifiable CRF is of great importance due to both their own health behaviors and their positions in health service delivery.

**Supplementary Information:**

The online version contains supplementary material available at 10.1186/s12909-025-07487-y.

## Introduction

Cancer is a disease in which some cells in the body grow uncontrollably and spread to different parts of the body [1]. There are more than a hundred types of cancer and cancer types are named according to the type of cell that forms cancer, such as epithelial cell or squamous cell, and the organs or tissues where cancers occur [1]. It was responsible for approximately 1 of every 6 deaths in the world in 2020 [2]. According to GLOBOCAN 2020 data, the incidence of cancer in the world was 19,292,789, its mortality was 9,958,133, and its five-year prevalence was 50,550,287. Lung, colorectal, liver, and stomach cancers were the most common types of cancer that caused death in 2020 [2]. Cancers with the highest incidence are breast, lung, colorectal and prostate cancers [2]. It is expected that 1,918,030 new cases and 609,360 cancer-related deaths will occur in 2022 in the USA [3]. Moreover, it is expected that new cases will be most frequently caused by breast, prostate, lung and colorectal cancers, and the most common death will be from lung, colorectal, pancreatic and breast cancer [3]. The lifetime risk of developing cancer has been reported as 39.9% [3]. In 2020, there were 233,824 reported cancer cases in Turkey, with the most common ones being breast, prostate, lung, and colorectal cancers. The cancer mortality number was 126,335, the most common cancers causing death were lung, breast, prostate, and stomach [2].

Approximately one third of cancer deaths are caused by tobacco use, obesity, alcohol consumption, malnutrition, and lack of physical activity [4]. Cancer-causing infections such as human papillomavirus (HPV) and hepatitis are responsible for approximately 30% of cancer cases in low- and middle-income countries [4]. It is argued that 30–50% of cancers can be prevented by being mindful about cancer risk factors (CRF), avoiding risk factors and applying evidence-based prevention strategies [4]. Cancer prevention may include maintaining a healthy lifestyle, avoiding cancer-causing substances, and using medicine or vaccines that can prevent cancer from developing [5]. Risk factors that a person can control are called modifiable risk factors.

Among the modifiable CRF, the most important ones are tobacco and alcohol use, obesity, HPV infection and malnutrition. It has been stated that smoking causes approximately 30% of all cancer deaths in the United States [5]. Smoking is strongly associated with an increased risk for many types of cancer (acute myeloid leukemia, kidney cancer, lung cancer, oral cancer,….) [5]. HPV increases the risk of penile, vaginal, anal, cervical, and oropharyngeal cancer.^5^ Other important CRFs such as alcohol use can cause mouth, colorectal, liver cancer and obesity can cause breast cancer, colorectal cancer, kidney cancer [5].

Studies have found that high school and university students [6], teachers [7] and the general population [8] have lack of knowledge about CRF. Cancer is one of the most common causes of death worldwide, making it critically important for medical students to receive education on this topic [2]. Such education plays a pivotal role in early diagnosis, treatment, and prevention. The involvement of medical students in clinical practice during their training, as well as their role in primary healthcare delivery after graduation, underscores the significance of their knowledge about cancer risk factors. Understanding these risk factors enables future physicians to better assess their patients and provide more accurate guidance in their clinical practice. Although previous studies have assessed knowledge of CRFs in specific populations, this study is one of the few to comprehensively evaluate awareness of multiple modifiable CRFs across a broad sample of medical students, providing a more holistic understanding of educational gaps. In this study, we aimed to determine the medical students’ knowledge level of about CRF.

## Methods

This study was carried out between February 10 and March 04 of 2022 at Gaziantep University (public university), which is located in Gaziantep, a city in the Southeastern Anatolia Region of Turkey. Before the study, 1574 students at Gaziantep University Faculty of Medicine were informed about the study and 532 (33.8%) of them gave their consent and completed a survey. The sociodemographic data of the students and their level of knowledge about CRF were questioned in the survey conducted by face-to-face interview method.

The risk status of modifiable risk factors (smoking, alcohol, obesity, HPV, high-fat diet) for cancer types was questioned. For the risk factor suggested in the questions, the students were asked to give one of following answers: “increases the risk”, “reduces the risk”, “does not affect the risk”, and “I don’t know”. The answer “increases the risk’” was accepted as the correct answer and was given 1 point. Among the risk factors questioned, smoking was a risk factor for 11 cancer types, alcohol for 7, obesity for 9, HPV for 5, and high-fat diet for 4. For a total of 36 questions, the level of knowledge was evaluated over 36 points. For the question examining sources of knowledge about CRFs, participants were allowed to select multiple options. The American cancer society guideline was taken as a reference in the determination of CRFs [9]. This survey was created for this study and has not been published anywhere. The reliability of knowledge questions on CRF was assessed using the Cronbach α internal consistency coefficient. The Cronbach alpha value was 0.840.

Additionally, multivariate linear regression analysis was performed to identify independent predictors of knowledge score. Predictor variables included gender, age, academic year, prior training on CRF, and having a family member with cancer.

Permission for this study was obtained from Gaziantep University Faculty of Medicine Scientific Research Ethics Committee (Date: 26 January 2022, No: 2022/45) and Gaziantep University Faculty of Medicine Dean’s Office (Date: 18 January 2022, No: 139809).

### Statistical analysis

Statistical analysis was performed using SPSS 23.0. Frequency, percentage, mean and standard deviation values were used in the analysis of the data. The conformity of the variables to the normal distribution was verified with Kolmogorov-Smirnov and Shapiro-Wilk tests. Student t and Chi-square tests were used for pairwise group evaluations. The relationship between parametric variables and CRF knowledge level was analyzed using the Pearson correlation test. Cases with a p-value below 0.05 were considered statistically significant.

## Results

The students were between the ages of 17–48 with the mean age of 22.63 ± 2.66 years. Of the participants, 270 (50.8%) were female and 262 (49.2%) were male. Ninety-six students (18.0%) stated that at least one of their family members had been diagnosed with cancer. The sociodemographic data of the students are given in Table 1.

**Table 1:** Participants’ sociodemographic data and their knowledge scores about cancer risk factors

Parameter	Variable	*N* (%)	Total score (Mean ± SD)	*p*
Gender	Woman	270 (50.8)	23.06 ± 7.35	0.674
	Man	262 (49.2)	23.32 ± 7.08	
Year	Preclinical years (year 1–3)	207 (38.9)	21.09 ± 8.02	< 0.001
	Clinical years (year 4–6)	325 (61.1)	24.50 ± 6.30	
Marital Status	Married	16 (3.0)	25.43 ± 6.37	0.202
	Single	516 (97.0)	23.10 ± 7.22	
Do you have a family member that was diagnosed with cancer	Yes	96 (18.0)	24.29 ± 6.16	0.062
	No	436 (82.0)	22.92 ± 7.39	
Have you receive training related to CRFs?	Yes	242 (45.5)	25.12 ± 6.11	< 0.001
	No	290 (54.5)	21.54 ± 7.64	
Would you like to receive training related to CRFs?	Yes	450 (84.6)	23.09 ± 7.16	0.563
	No	82 (15.4)	23.59 ± 7.44	

* Cancer Risk Factors (CRF)

Among the participants, 242 (45.5%) stated that they received training on CRF, and 450 (84.6%) stated that they wanted to receive training. When asked about the source of CRF-related information that they had received 304 (57.1%) students stated that they received it from internet, 285 (53.6%) from medical education, 160 (30.1%) from their family and friends, 157 (29.5%) from TV programs, and 83 (15.6%) from healthcare workers.

The mean CRF knowledge score of the participants was 8.74 ± 2.91 for smoking, 4.81 ± 2.03 for alcohol, and 23.17 ± 7.20 for all risk factors. The knowledge scores of the students according to CRF are given in the Table 2. Although it varies according to the type of cancer, respondents indicated that smoking (69.0-96.6%), alcohol (49.1–85.9%), obesity (42.9–73.3%), HPV (26.5–65.2%), and high-fat diet (33.1–62.4%) as CRFs (Table 3).

**Table 2:** Distribution of student scores according to cancer risk factors

Risk factor	Knowledge score mean ± SD	Knowledge score min-max
Smoking	8.74 ± 2.91	0–11
Alcohol	4.81 ± 2.03	0–7
Obesity	5.15 ± 3.12	0–9
HPV	2.51 ± 1.48	0–5
High fat diet	1.91 ± 1.40	0–4
All risk factors	23.17 ± 7.20	0–36

**Table 3:** Responses of students to questions regarding cancer risk factors

Cancer risk factor	Cancer type	Increases risk *N* (%)	Reduces risk *N* (%)	Does not affect *N* (%)	I don’t know *N* (%)
Smoking	Lung	514 (96.6)	5 (0.9)	8 (1.5)	5 (0.9)
	Mouth	475 (89.3)	4 (0.8)	13 (2.4)	40(7.5)
	Stomach	438 (82.3)	17 (3.2)	27 (5.1)	50 (9.4)
	Kidney	427 (80.3)	4 (0.8)	24 (4.5)	77 (15.5)
	Liver	417 (78.4)	6 (1.1)	27 (5.1)	82 (15.4)
	Cervical	415 (78.0)	6 (1.1)	38 (7.1)	73 (13.7)
	Head-neck	407 (76.5)	7 (1.3)	33 (6.2)	85(16.0)
	Pancreatic	406 (76.3)	6 (1.1)	35 (6.6)	85 (16.0)
	Colorectal	404 (75.9)	12 (2.3)	30 (5.6)	86 (16.2)
	Skin	383 (72.0)	5 (0.9)	50 (9.4)	94 (17.7)
	Anal	367 (69.0)	6 (1.1)	52 (9.8)	107 (20.1)
Alcohol	Liver	457 (85.9)	4 (0.8)	21 (3.9)	50 (9.4)
	Stomach	438 (82.3)	4 (0.8)	36 (6.8)	54 (10.2)
	Pancreatic	402 (75.6)	3 (0.6)	45 (8.5)	82 (15.4)
	Colorectal	376 (70.7)	3 (0.6)	62 (11.7)	91 (17.1)
	Mouth	346 (65.0)	4 (0.8)	80 (15)	102 (19.2)
	Breast	283 (53.2)	2 (0.4)	111 (20.9)	136 (25.6)
	Head-neck	261 (49.1)	3 (0.6)	115 (21.6)	153 (28.8)
Obesity	Stomach	390 (73.3)	41 (7.7)	100 (18.8)	1 (0.2)
	Liver	367 (69.0)	0 (0)	47 (8.8)	118 (22.2)
	Colorectal	363 (68.2)	0 (0)	51 (9.6)	118 (22.2)
	Pancreatic	333 (62.6)	1 (0.2)	69 (13.0)	129 (24.2)
	Renal	307 (57.7)	1 (0.2)	90 (16.9)	134 (25.2)
	Breast	257 (48.3)	3 (0.6)	114 (21.4)	158 (29.7)
	Mouth	256 (48.1)	4 (0.8)	120 (22.6)	152 (28.6)
	Prostate	237 (44.5)	4 (0.8)	120 (23.1)	152 (31.6)
	Head-neck	228 (42.9)	0 (0)	127 (23.9)	177 (33.3)
HPV	Cervical	453 (65.2)	3 (0.6)	17 (3.2)	58 (10.9)
	Skin	312 (58.6)	1 (0.2)	87 (16.4)	132 (24.8)
	Anal	222 (41.7)	3 (0.6)	132 (24.8)	175 (32.9)
	Mouth	212 (39.8)	3 (0.6)	130 (24.4)	187 (35.2)
	Head-neck	141 (26.5)	2 (0.4)	183 (34.4)	206 (38.7)
High Fat Diet	Colorectal	332 (62.4)	3 (0.6)	62 (11.7)	135 (25.4)
	Pancreatic	328 (61.7)	4 (0.8)	64 (12.0)	136 (25.6)
	Prostate	184 (34.6)	3 (0.6)	129 (24.2)	216 (40.6)
	Breast	176 (33.1)	4 (0.8)	167 (31.4)	185 (34.8)

The CRF-related knowledge score of women was 23.06 ± 7.35 while for men it was 23.32 ± 7.08. Knowledge scores of women and men were similar (*p* = 0.674). The level of knowledge about CRF was found to be significantly lower in preclinical (1-3rd year) students than in clinical (4-6th year) students (*p* < 0.001). There was a significant correlation between age and knowledge score (*p* < 0.001, *r* = 0.287). Similarly, a significant correlation was observed between class level and knowledge score (*p* < 0.001, *r* = 0.274). The knowledge score of those who received training on CRF was found to be 25.12 ± 6.11, while it was 21.54 ± 7.64 in those who did not. This difference was significant (*p* < 0.001). Students’ CRF knowledge scores are presented in the table (Table 1).

In the multivariate regression model, being in clinical years (Beta = 0.34, *p* < 0.001), receiving CRF training (Beta = 0.28, *p* < 0.001), and increasing age (Beta = 0.12, *p* = 0.01) were positively associated with higher CRF knowledge scores, whereas gender and family history of cancer were not significant predictors. The model explained 32.4% of the variance in knowledge scores (Adjusted R² = 0.324).

Among the students, 514 (96.6%) stated that smoking was a risk factor for lung cancer, 475 (89.3%) mouth cancer, and 438 (82.3%) stomach cancer. In cancer types where alcohol use is a risk factor, the students most frequently stated liver (*n* = 457, 85.9%) and stomach cancer (*n* = 438, 82.3%). Of the students, 390 (73.3%) considered obesity as a risk factor for stomach cancer, 367 (69.0%) for liver cancer, and 363 (68.2%) for colorectal cancer. Among the cancers for which HPV is a risk factor, the most commonly known cancers were cervical (*n* = 453, 65.2%) and skin cancer (*n* = 312, 58.6%), while the least known ones are head and neck (*n* = 141, 26.5%) and mouth cancer (*n* = 212, 39.8%. Lastly, 332 (62.4%) students stated that high-fat diet is a risk factor for colorectal cancer. Responses to CRF are indicated in the Table 3.

## Discussion

This study found that the overall mean knowledge score about cancer risk factors among medical students was 23.17 out of 36, highlighting a considerable gap in awareness. Female and male students scored similarly, and scores were significantly higher among students in clinical years, those with prior training, and older students. In this study, the knowledge scores of men and women about CRF were analogous (*p* = 0.674). In a study conducted in Malaysia, no relationship between the level of CRF knowledge of medical students and their gender was found [10]. The knowledge level about colorectal CRF were similar in men and women who were university students studying medical sciences in Jordan [11] and medicine in Saudi Arabia [12]. The level of knowledge of medical students on breast cancer risk factors in Syria was found to be similar in men and women [13]. In a study conducted among medical students in Turkey, the level of knowledge regarding cancers caused by HPV was found to be similar between female and male participants [14]. Our study’s findings were similar to the literature. This might be because medical students undergo similar educational process that is not gender-based.

The CRF-related knowledge was significantly lower in preclinical students than in clinical students (*p* < 0.001). The knowledge score of those who received training on CRF was significantly higher than those who did not (*p* < 0.001). In this study, being in clinical years and having received prior training were the strongest predictors of CRF knowledge. These findings align with previous literature and underscore the importance of formal training in raising awareness. In this study, a significant positive correlation was observed between the medical school class level and knowledge of CRF. In this study, being in clinical years and having received prior training were the strongest predictors of CRF knowledge. These findings align with previous literature and underscore the importance of formal training in raising awareness. In a study conducted with medical students in Syria, the level of knowledge regarding oral cancer risk factors was found to be significantly higher in clinical term students than that of preclinical students [15]. There was a significant correlation between the oral CRF knowledge level and the academic year of the dental students in Romania [16]. In a study conducted by Seckiner et al. among medical students in Turkey, it was found that the level of knowledge regarding cancers caused by HPV was significantly higher among clinical-term students compared to preclinical-term students [14]. In our study and in the literature, the level of knowledge about CRF of preclinical (1-3rd grade) students was found to be lower than that of clinical students. This maybe because medical students encounter cancer patients during the clinical period. In addition, the high level of knowledge of those trained on CRF indicates that trainings are beneficial in increasing the CRF knowledge.

In our study, 45.5% of participants stated that they received training on CRF and 84.6% stated that they wanted to receive such training. Respondents stated that most frequently they obtained information about CRF from the internet (57.1%) and medical education (53.6%). Medical students in Turkey mostly benefit from medical education and the internet as a source of information on risk factors for childhood cancers [17]. Our study shows that medical students do not receive adequate training on CRF. An important finding of our study is that a significant portion of medical students want to receive such training. Our study and the literature show that medical students most frequently obtain information about CRF from medical education and the internet. We believe that providing medical education on CRF as a standard to all medical students and organizing special modules for medical students on CRF and medical education over the internet will be of great benefit.

In this study, smoking and alcohol use were the most frequently reported CRFs. This may be because smoking and alcohol have a big place in the daily flow of our lives and research on these topics is extensive.

In this study, 96.6% of the students stated that smoking was a risk factor for lung cancer and 89.3% for oral cancer. In another study conducted with medical students, 88.3% of the students stated that smoking is a risk factor for oral cancer [10]. In a study conducted with university students, 99% of participants stated that smoking is a risk factor for lung cancer [18]. In our study and in the literature, very high rate of students stated that smoking is a risk factor, especially for lung and mouth cancers. The effects of smoking on health are introduced to health workers and the society with many different methods and practices. Despite this, it is remarkable that it is not known to a great extent which cancer types it is a risk factor for. The training and practices given to medical students on this subject need to be done more effectively.

The students stated that alcohol use was the most common risk factor for liver cancer (85.9%) and stomach cancer (82.3%). In a study by Merten et al. conducted with university students, 86% of the students stated alcohol as a risk factor for liver cancer and 60% for colorectal cancer [18]. It was stated by the students in our study and Merten et al.‘s study that alcohol use is a risk factor especially for liver cancer. It was notable that medical students did not know that alcohol use is a risk factor for some other cancer types as well. Medical students need to know the effects of alcohol on cancer due to their individual health behavior and their place in health service delivery.

In our study, 73.3% of the students considered obesity as a risk factor for stomach cancer, 69.0% for liver, and 68.2% for colorectal cancer. In a Polish study, 66.8% of medical students considered obesity as a risk factor for colorectal cancer [19]. In a study conducted with university students in the USA, more than half of the students stated that obesity is a risk factor for colorectal cancer [18]. In similar studies, knowledge about obesity being a risk factor for colorectal cancer seems to be at the forefront compared to other cancer types. This may be due to the belief that obesity is related to increased risk of colorectal cancer. In addition, the fact that some types of cancer, for which obesity is a risk factor, were inquired while others were not, may have led to this situation. Obesity is a problem whose impact on health is increasing day by day worldwide. It is important that obesity is a risk factor for many types of cancers and medical students’ knowledge about this situation will contribute to preventive health policies.

Among the cancers for which HPV is a risk factor, the most commonly known cancers were cervical (65.2%) and skin cancer (58.6%), while the least known ones were head and neck (26.5%) and mouth cancer (39.8%). In a study conducted in Malaysia, 53.7% of medical students stated HPV as a risk factor for oral cancer [10]. In Jordan, 84.8% of medical students stated that HPV poses a risk for cervical cancer and 21.0% for oropharyngeal cancer [20]. In a study conducted with university students in the USA, 82% of the students noted HPV as a risk factor for cervical cancer and 24% for head and neck cancer [18]. In a study conducted among medical students in Turkey, 82.9% of the participants reported that HPV causes cervical cancer, while 57.3% stated that it is a cause of oral cancer [14]. Our study and the literature show that a high percentage of students know that HPV is a risk factor for cervical cancer. However, knowledge that HPV is a risk factor for oral cancer was not as prevalent among medical students and it is a finding that needs to be investigated. Our study is in agreement with the literature.

Among the students, 62.4% stated that a high-fat diet is a risk factor for colorectal cancer. In Poland, 83.7% of medical students stated that a high-fat diet is a risk factor for colorectal cancer [19]. Our results are similar to the literature. Diet can be a risk factor and protective factor for a wide variety of health problems. It was more widely known by students that high-fat diet is a risk factor for colorectal cancer.

In this study highlights the need to structure medical education curricula to enhance awareness of CRFs. In this context, adopting multidisciplinary approaches within the curriculum is essential. Additionally, case-based learning methods and simulation-based applications can better prepare students for real-life situations. To foster critical thinking skills, interactive discussion environments and sessions analyzing current literature on CRFs should be incorporated. Finally, periodic evaluation mechanisms should be established to assess the effectiveness of these strategies, enabling continuous improvement based on the results. These recommendations aim to facilitate the effective and evidence-based improvement of the curriculum.

Compared to earlier studies focusing on single cancer types or risk factors, our study’s holistic approach to evaluating knowledge across multiple CRFs and cancer types provides a broader perspective and allows for better curriculum planning. This discussion highlights the need to revise medical education programs to systematically integrate cancer prevention and risk factor awareness, supported by regular assessments and case-based learning to reinforce key messages.

### Limitations and strengths of the study

This study was conducted exclusively with medical students from a single university. Therefore, the findings are limited to this institution and may not be generalizable to the broader population of medical students. The survey’s focus on questions solely formatted to test correct knowledge of risk factors might limit its ability to deeply assess students’ comprehensive understanding of these factors. However, this study could be the first in the literature to holistically evaluate medical students’ knowledge of risk factors for various cancers. The large sample size and the internal consistency of the survey, measured by a Cronbach’s alpha value of 0.840, indicate the reliability of the study.

## Conclusion

It was determined that medical students had serious lack of knowledge about CRF. The knowledge level of men and women about CRF was similar. Education positively impacted students’ CRF knowledge and medical education curricula need to be further developed in this area. Students’ knowledge about smoking and alcohol being CRF were at a higher rate than other risk factors. Educating students about modifiable CRF is of great importance due to both their own health behaviors and their positions in healthcare. Our findings indicate that while knowledge on smoking and alcohol as CRFs is relatively high, awareness of other risk factors like HPV and obesity is lacking. Regression analysis confirmed the importance of clinical exposure and targeted education in improving scores. We recommend revising medical curricula to incorporate standardized, comprehensive education on modifiable cancer risk factors.

## Electronic supplementary material

Below is the link to the electronic supplementary material.

Supplementary Material 1

## Data Availability

No datasets were generated or analysed during the current study.

## Abbreviations

HPV: Human Papillomavirus.
CRF: Cancer Risk Factors.
