# Supplementary material for: How much do medical students know about cancer risk factors?
Source: BMC Med Educ. 2025 Jul 1;25:904. doi: 10.1186/s12909-025-07487-y (PMC12211475; doi:10.1186/s12909-025-07487-y)
Supplement: Supplementary file 1 — Supplementary Material 1 [file 12909_2025_7487_MOESM1_ESM.docx]

| Cancer risk factor | Cancer type | Increases risk | Reduces risk | Does not affect | I don’t know |
| --- | --- | --- | --- | --- | --- |
| Smoking | Lung |  |  |  |  |
|  | Mouth |  |  |  |  |
|  | Stomach |  |  |  |  |
|  | Kidney |  |  |  |  |
|  | Liver |  |  |  |  |
|  | Cervical |  |  |  |  |
|  | Head-neck |  |  |  |  |
|  | Pancreatic |  |  |  |  |
|  | Colorectal |  |  |  |  |
|  | Skin |  |  |  |  |
|  | Anal |  |  |  |  |
|  |  |  |  |  |  |
| Alcohol | Liver |  |  |  |  |
|  | Stomach |  |  |  |  |
|  | Pancreatic |  |  |  |  |
|  | Colorectal |  |  |  |  |
|  | Mouth |  |  |  |  |
|  | Breast |  |  |  |  |
|  | Head-neck |  |  |  |  |
|  |  |  |  |  |  |
| Obesity | Stomach |  |  |  |  |
|  | Liver |  |  |  |  |
|  | Colorectal |  |  |  |  |
|  | Pancreatic |  |  |  |  |
|  | Renal |  |  |  |  |
|  | Breast |  |  |  |  |
|  | Mouth |  |  |  |  |
|  | Prostate |  |  |  |  |
|  | Head-neck |  |  |  |  |
|  |  |  |  |  |  |
| HPV | Cervical |  |  |  |  |
|  | Skin |  |  |  |  |
|  | Anal |  |  |  |  |
|  | Mouth |  |  |  |  |
|  | Head-neck |  |  |  |  |
|  |  |  |  |  |  |
| High Fat Diet | Colorectal |  |  |  |  |
|  | Pancreatic |  |  |  |  |
|  | Prostate |  |  |  |  |
|  | Breast |  |  |  |  |
